# Efficacy and Safety of Fulvestrant 500mg in Hormone-receptor Positive Human Epidermal Receptor 2 Negative Advanced Breast Cancer: A Real-world Study in China

**DOI:** 10.7150/jca.47960

**Published:** 2020-09-23

**Authors:** Wen Lei, Huiping Li, Guohong Song, Ruyan Zhang, Ran Ran, Ying Yan, Lijun Di, Hanfang Jiang

**Affiliations:** Key Laboratory of Carcinogenesis and Translational Research (Ministry of Education/ Beijing), Department of Breast Oncology, Peking University Cancer Hospital and Institute, Beijing 100142, China

**Keywords:** Advanced breast cancer, Endocrine therapy, Fulvestrant, hormone-receptor positive

## Abstract

**Background:** Fulvestrant 500mg has proved its clinical effectiveness in previous trials as primary or second line treatment of hormone receptor positive, human epidermal receptor 2 negative (HR+/HER2˗) post-menopausal advanced breast cancer. This real-world study aimed to investigate the efficacy and safety of Fulvestrant in HR+/HER2˗ Chinese advanced breast cancer patients.

**Method:** HR+/HER2˗ advanced breast cancer patients who received Fulvestrant 500mg from January 2015 to December 2018 in Beijing Cancer Hospital were enrolled in this retrospective study. Progression free survival (PFS), objective response rate (ORR), clinical benefit rate (CBR), overall survival (OS) and adverse events (AEs) of Fulvestrant were investigated.

**Result:** In total 303 enrolled patients [median age was 51 years (range: 21-82)], 255 (84.2%) patients were at postmenopausal status at the start of Fulvestrant treatment and 264 patients (87.1%) had advanced breast cancer. The median PFS (95% confidence interval) was 14.1 months (10.1-18.0) for the first-line, 11.2 months (2.2-20.3) for the second-line and 6.7 months (4.8-8.5) for ≥third-line of Fulvestrant. The ORR and CBR were 3.8% and 86.8% for the first-line, 5.5% and 75.4% for the second-line, 1.1% and 61.1% for ≥third-line of Fulvestrant. The multivariate subgroup analyses showed, PFS was significantly longer for the patients with light tumor burden, less palliative chemotherapy before Fulvestrant and long disease-free interval. For patients receiving Fulvestrant after palliative chemotherapy, the median PFS was numerically greater in maintenance treatment group than those who progressed after chemotherapy. Only 5.0% of patients (15/303) experienced adverse events and majority were grade 1-2. The most common adverse event was headache and palpitation, with merely one patient had severe adverse event (pulmonary embolism).

**Conclusion:** Fulvestrant is an effective, safe and well-tolerated treatment regimen in endocrine therapy for HR+/HER2˗ metastatic breast cancer. Light tumor burden, less palliative chemotherapy before Fulvestrant and long disease-free survival (DFS) might be the ideal condition of Fulvestrant treatment. Fulvestrant can be effective for premenopausal patients with drug-induced menopause. Patients of different luminal subtypes can benefit from Fulvestrant. For patients with visceral metastases, presence of liver metastases rather than lung metastases was poor prognostic factor. Fulvestrant may also be considered as a maintenance treatment after first-line palliative chemotherapy.

## Introduction

Breast cancer (BC) ranks the most common cancer among women worldwide. In China, it was estimated that 405680 female patients are prone to breast cancer by 2025^1^. With the progression of diagnosis and treatment recently, the prognosis of breast cancer population has improved significantly. However, breast cancer is a heterogeneous tumor, and there are certain differences in the treatment and prognosis of different breast cancer subtypes. Around 60 - 70% of advanced BC have hormone receptor (HR) positive, human epidermal receptor 2 (HER2) negative (HR+/HER2˗) tumors^2^, ^3^ , which is the hotspot of research.

Due to the expression of hormone receptors, endocrine therapy has become an important strategy for this subtype of breast cancer^4^. And because maintaining the quality of life is an important treatment goal for advanced breast cancer^5^, international guidelines recommend endocrine therapy as the first-line therapy for HR+/HER2˗ advanced BC patients if these patients didn't have life-threatening disease, symptomatic visceral metastasis, or had resistance to prior endocrine therapy^6^-^8^. In addition to traditional selective estrogen receptor modulators (SERMs) and aromatase inhibitors (AIs), the emergence of Fulvestrant has added new treatment option for endocrine therapy. Fulvestrant, a selective estrogen receptor down-regulator (SERD), exhibits the characteristics of high affinity, complete inhibition of estrogen receptor (ER) nuclear translocation and without estrogenic effects^9^-^11^. At present, a series of clinical studies have determined the dose of fulvestrant 500mg 12-15, and other clinical studies comparing AIs have confirmed the efficacy and safety of Fulvestrant 500mg in HR+/HER2˗ advanced breast cancer^16^-^20^. Among them, the phase III FALCON trial which demonstrated Fulvestrant 500mg has a significant advantage over AIs in PFS (16.6 months vs 13.8 months), becomes an important basis for the choice of first-line endocrine therapy for HR+ advanced breast cancer post-menopause patients^17^. And the efficacy of Fulvestrant in the visceral metastasis subgroup in the study failed to show advantage over AIs^17^. Another clinical study have reached a similar conclusion that presence of visceral metastasis was a risk factor for the efficacy of fulvestrant^21^. On the contrary, a meta-analysis comparing Fulvestrant therapy with aromatase inhibitors by Graham et al showed that women with visceral metastasis derive higher benefits from Fulvestrant treatment^22^.

Despite the substantial evidence suggesting the efficacy of Fulvestrant by randomized control trials (RCTs), there is paucity of available literature regarding the safety and effectiveness of Fulvestrant 500mg in the real-world settings of patients with advanced breast cancer. The study population of current real-world data, which is different from those Chinese patients with characteristic of younger age of diagnosis, higher proportion of premenopausal and primary stage IV breast cancer, cannot well represent the actual situation of China^23^-^26^. In addition, the research concerning Chinese breast cancer population has the limitation of small sample size^27^. Therefore, the present retrospective study is conducted to assess the efficacy and safety of Fulvestrant 500mg by larger sample in real-world settings and to assess the risk factors affecting Fulvestrant 500mg among Chinese HR+/HER2˗ advanced breast cancer patients.

## Materials and Methods

### Study design and patient population

This real-world study was conducted at the department of breast oncology in Beijing Cancer Hospital between January 2015 to December 2018.

**Fig 1:**
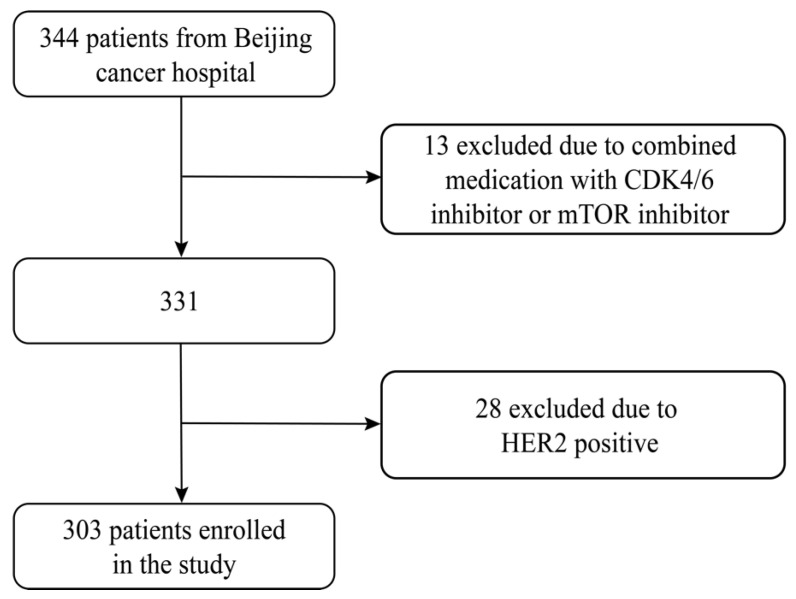
Flowchart of this study

The inclusion criteria were women with histologically confirmed HR positive (ER positive and/or PgR positive) advanced breast cancer and received at least once evaluation during Fulvestrant 500mg treatment. Fulvestrant was administered on days 1,15 and every 28 days thereafter. Key exclusion criteria were HER2 positive, combined medication with cyclin-dependent kinase (CDK) 4 and 6 inhibitors, mammalian target of rapamycin (mTOR) inhibitor or chemotherapy. The ovarian function suppression was allowed on the condition that the patient was premenopausal. In total, 303 patients were included and excluding HER2 positive patients (n=28) and combined medication with CDK 4/6 inhibitor or mTOR inhibitor (n=13) (Figure 1).

Fulvestrant was administered intramuscularly at a dose of 500mg on days 1, 15, and every 28 days thereafter, as two 5mL injections at each buttock.

### Definitions

Patients receiving Fulvestrant who had not received any chemotherapy or endocrine therapy for metastatic disease were considered first-line patients. Patients receiving Fulvestrant as initial treatment for metastatic disease or progression after palliative chemotherapy were considered first-line endocrine therapy. Primary endocrine resistance was defined as relapse while on the first 2 years of adjuvant endocrine therapy, or progression disease (PD) within first 6 months of first-line endocrine therapy for advanced disease. Secondary endocrine resistance was defined as relapse while after the first 2 years of adjuvant endocrine therapy, or relapse within 12 months of completing adjuvant endocrine therapy, or PD ≥ 6 months after initiating endocrine therapy for advanced disease^28^.

### Efficacy and safety endpoints evaluation criteria

The efficacy of treatment was evaluated in accordance with the Response Evaluation Criteria In Solid Tumors (RECIST) version 1.1^29^. Clinical benefit rate (CBR) was defined as the proportion of patients experiencing complete response (CR), partial response (PR) or stable disease (SD). ORR was defined as the proportion of patients with CR or PR. Progression free survival (PFS) was defined as the time from the beginning of Fulvestrant treatment to progression or death from any cause. Overall survival (OS) was defined as the time from Fulvestrant initiation to death from any cause.

Adverse events were evaluated using the National Cancer Institute Common Toxicity Criteria (CTCAE), Version 4.0.

### Statistical analysis

SPSS 23.0 was used for all statistical analyses. Estimation of survival was performed using the Kaplan-Meier method, and differences between survival curves were assessed with the log-rank test. Univariate and multivariate analyses with Cox proportional hazards regression models were used to identify independent prognostic factors. A two-sided *p* value<0.05 was considered significant.

## Results

### Baseline characteristics of the study population

In total, 303 patients were enrolled in this study. The median age was 51 years (range: 21-82). 255 (84.2%) patients were at postmenopausal status at the start of Fulvestrant treatment. The majority of the patients (264, 87.1%) had advanced breast cancer after radical surgery and 12.9% patients had primary stage IV breast cancer. More than three quarters of patients (77.6%) were diagnosed with invasive ductal carcinoma. The patients who showed high expression in HR (≥50%) were 70.3% and 212/264 (80.3%) patients had previously received adjuvant endocrine therapy. Around half of our patients (50.5%) had received AIs during adjuvant treatment. Sociodemographic and disease characteristics of the patients are presented in Table 1.

### Metastases status and treatment

Visceral metastases at the treatment of Fulvestrant was observed among 68.3% of the patients with bone (72.3%) being the most common metastatic site and 35.0% of the total patients didn't receive chemotherapy before Fulvestrant for metastatic breast cancer disease. Fulvestrant as the first-line, second-line and ≥ third-line of treatment was received by 53 (17.5%), 73 (24.1%), and 177 (58.4%) of the patients, respectively. Fulvestrant as first-line, second-line and ≥ third-line endocrine therapy was initiated by 31.7%, 40.6%, and 27.7% of the patients, respectively. Out of 96 patients treated with Fulvestrant as first-line endocrine therapy, 55.2% initiated without chemotherapy, 30.2% initiated after progression of previous chemotherapy and 14.6% patients received as maintenance treatment after chemotherapy (Table 2).

### Efficacy outcomes

Out of 303 patients 2.6% reached PR, 69.3% were evaluated as SD, 28.1% evaluated as PD. The ORR and CBR were 3.8% and 86.8% for the first-line, 5.5% and 75.4% for the second-line, 1.1% and 61.1% for ≥ third-line of Fulvestrant treatment.

**Fig 2:**
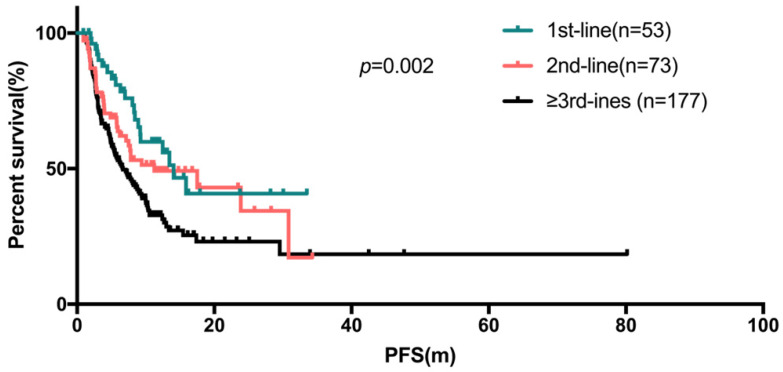
The Kaplan-Meier curve for PFS stratified by treatment line of fulvestrant

The median PFS (95% confidence interval) were 14.1 months (10.1 - 18.0), 11.2 months (2.2 - 20.3), 6.7 months (4.8 - 8.5) for the first-line, second-line and ≥ third-line of Fulvestrant respectively (Figure 2), compared to 8.5 months (7.0-10.0) for all patients.

### Predictors of risk factors

**Fig 3:**
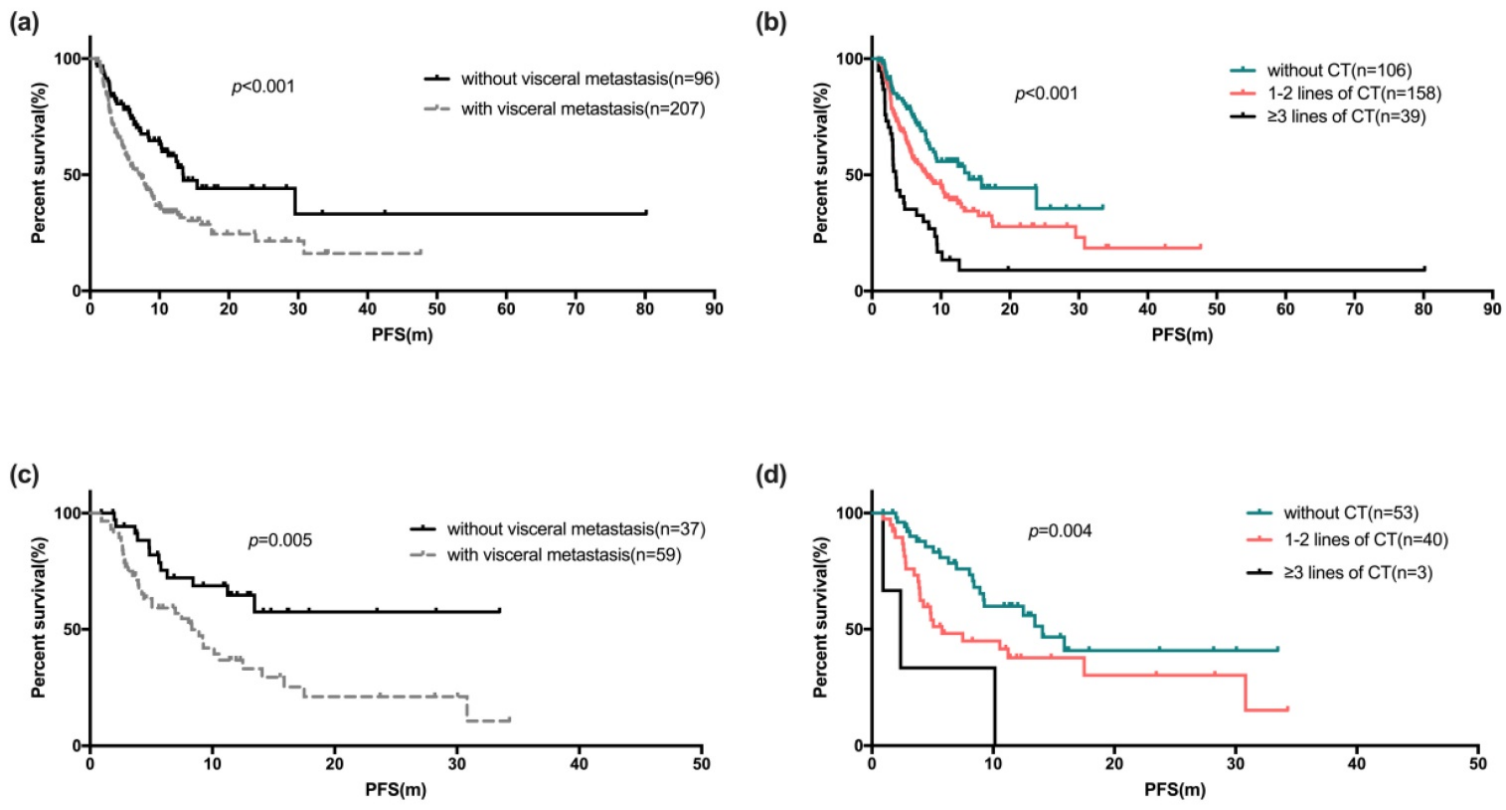
The Kaplan-Meier curve for PFS stratified by visceral metastases (a) and palliative chemotherapy before fulvestrant (b). The Kaplan-Meier curve of PFS of Fulvestrant used as first-line endocrine therapy stratified by visceral metastases (c) and palliative chemotherapy before fulvestrant (d)

Univariate and COX regression analyses showed factors like patients with age ≥50 years (*p*=0.004), with no visceral metastases (*p*<0.001, Figure 3a), no liver metastases (*p*<0.001) and no chemotherapy for metastatic disease before Fulvestrant (*p*<0.001, Figure 3b) showed statistically significant difference (Table 3). Statistically significant difference in median PFS was also observed between the line of treatment of Fulvestrant (*p*=0.002) and the number of metastatic sites. However, statistically significant difference was not observed among the PFS in subgroup of patients with menopause status, HR status and Ki67 index. The COX regression model also showed that, the median PFS of Fulvestrant was significantly associated with DFS (*p*=0.027), number of metastatic sites (*p*<0.001), and previous chemotherapy for metastatic breast cancer (*p*=0.003). The median OS was not observed due to insufficient survival data.

In the first-line setting, the median PFS of those 53 patients (17.5%) was 14.1 months (95% CI: 10.1-18.0 months, range: 0.9-33.5 months). The subgroup analyses indicated that endocrine sensitive status was associated with a reduced risk of PFS compared to primary or secondary resistant to endocrine therapy (*p*<0.001, median PFS: not reached vs 3.7 vs 15.9 months). In the second-line setting, 31 of 73 patients received first-line chemotherapy another 42 patients received first-line endocrine therapy. After statistical analyses, there was no significant difference in the median PFS of Fulvestrant in the second-line patients after first-line chemotherapy or endocrine therapy (*p*=0.623).

**Fig 4:**
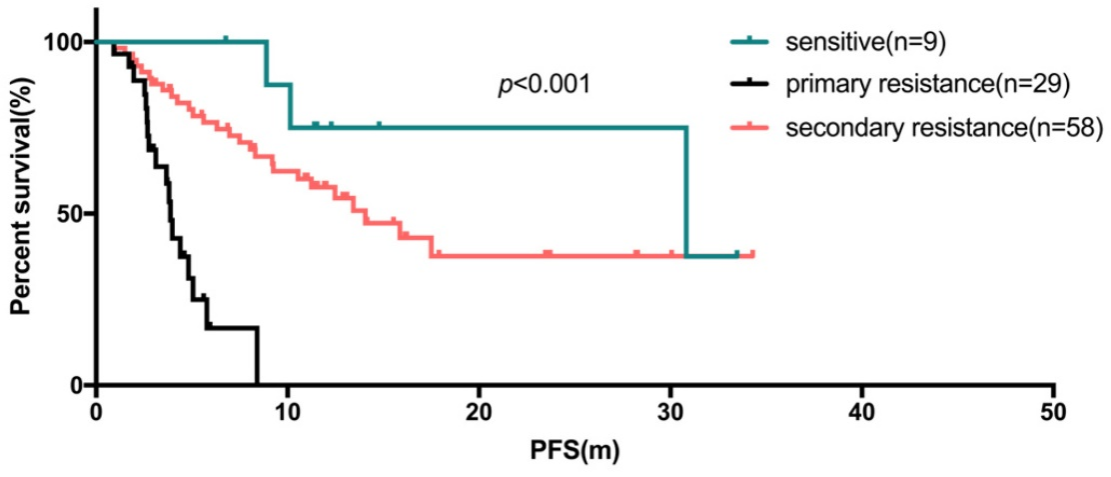
The Kaplan-Meier curve for PFS of fulvestrant used as first-line endocrine therapy stratified by sensitivity of endocrine therapy

There were 96 of 303 patients (31.7%) who initiated Fulvestrant as first-line endocrine therapy, either as initial therapy for metastatic disease, or after progression following chemotherapy or maintenance treatment after chemotherapy. Of these 96 patients, the median PFS was 10.5 months (95% CI: 6.5-14.6 months, range: 0.9-34.3 months). Table 4a shows univariate and COX regression analyses. Visceral metastases and prior palliative chemotherapy showed significant risk effect on PFS for Fulvestrant users (Figure 3c, 3d), and resistance to endocrine therapy was also a risk factor for poor PFS (Figure 4). These findings were supported by the COX regression analyses. The median PFS of patients receiving Fulvestrant as maintenance treatment after first-line chemotherapy was found to be numerically greater than that of patients receiving Fulvestrant after the progression of first-line chemotherapy (10.6 vs 4.9 months, *p*=0.356). For the patients who received Fulvestrant as first-line treatment or maintenance treatment after first-line chemotherapy, the median PFS of first-line treatment was found to be significantly longer than patients who progressed in first-line chemotherapy (14.1 vs 16.7 vs 8.6 months, *p*=0.044).

The majority of the patients (68.3%) were diagnosed with visceral metastases when receiving Fulvestrant. The COX regression analyses revealed that disease-free interval, numbers of metastatic sites, liver metastases were significant factors for PFS in the Fulvestrant users with visceral metastases. However, presence of lung metastases made no significant difference (Table 4b).

### Safety outcomes

In total, 15 (4.95%) adverse events occurred among the enrolled patients. Most of them (14/15; 93.3%) were mild or moderate in severity (grade 1 or 2), with the exception of one patient (0.33%) who had grade 3 pulmonary embolism. The most frequently observed adverse events were headache (n=2, 0.66%) and palpitation (n=2, 0.66%) (Table 5).

## Discussion

In this retrospective, observation real-world study, we analyzed the efficacy and safety in patients with HR+/HER2˗ advanced breast cancer treated with Fulvestrant monotherapy, and assessed the factors influencing the treatment response. Overall, the study results suggest that the PFS of patients receiving Fulvestrant as first-line palliative treatment was significantly longer than second-line and later-line treatment. Also, PFS was found to be significantly longer for the patients with light tumor burden, less palliative chemotherapy before Fulvestrant and long disease-free interval.

To our knowledge, Fulvestrant is recommended to be the standard option for first-line endocrine therapy in HR-positive advanced breast cancer patients. The outcome of phase II FIRST study (CBR: 72.5%; median PFS: 23.4 months)^20^, ^30^ and phase III FALCON study (CBR:78%; median PFS: 16.6 months)^24^ implied that Fulverstrant was more effective than third-generation aromatase inhibitors in patients without previous endocrine therapy. The real-world study from China and US reported that the PFS of Fulvestrant in first-line users were 15.67 months and 12.2 months respectively^24^, ^31^. However, in our study, only 17.5% (53/303) patients received Fulvestrant as the first-line treatment. The median PFS of patients receiving Fulvestrant as first-line treatment was significantly longer than second-line and later-line (*p*=0.002, 14.1, 11.2, 6.7 months respectively). This finding further implies that, the time to initiation of Fulvestrant treatment were essential factors influencing the treatment response. A multicenter retrospective study of 1072 patients with advanced/metastatic breast cancer who received Fulvestrant 500 mg in Japan reported by H. Kawaguchi et al. also concluded the use of Fulvestrant in the earlier line was more effective through multivariate analysis (HR=0.80, *p*<0.001) 32.

Furthermore, in our study the superiority of PFS in patients receiving fulvestant in the first-line setting over Fulvestrant first-line endocrine line users was numerically greater (14.1 vs 10.5 months, respectively). This result suggested that the prior chemotherapy affects efficacy of Fulvestrant, and this finding was supported by COX regression analyses. In which, patients receiving Fulvestrant as first-line endocrine therapy after palliative chemotherapy, the median PFS since Fulvestrant treatment was numerically greater in maintenance treatment group than those who progressed after first-line chemotherapy (10.6 vs 4.9 months, *p*=0.356). For PFS in first-line therapy, receiving Fulvestrant as first-line therapy and maintenance treatment after first-line chemotherapy were significantly better than those used after first-line chemotherapy progresses (14.1 vs 16.7 vs 8.6 months, *p*=0.044). This is a relatively new perspective because maintenance with endocrine therapy after chemotherapy is a considerable treatment pattern especially when the expected benefit of continuous chemotherapy is limited or the toxicity is unbearable. A small number of previous studies supported this treatment pattern, but none of them mainly used Fulvestrant as a maintenance drug^33^. A retrospective study investigating the efficacy of maintenance hormone therapy showed that median PFS of maintenance hormone therapy was 14.4 months (95% CI: 11.6-17.3 months), but 98.5% of the enrolled patients received AI or SERMs as maintenance therapy instead of Fulvestrant^34^. A prospective phase 2 study evaluating efficacy of Fulvestrant as maintenance therapy in Chinese patients with disease control after first-line chemotherapy illustrated that median PFS since Fulvestrant treatment was 16.1 months (95% CI: 10.3 - not reached), and median PFS since first-line chemotherapy was 19.5 months (95% CI: 15.6 - not reached)^35^. The median PFS obtained in our study was poorer than that in this prospective study, which may be due to effect of different chemotherapy regimen and population characteristics. Hence its noteworthy to believe that, as with the first-line use of Fulvestrant, the maintenance therapy of Fulvestrant after first-line chemotherapy is a very promising treatment strategy which also needs to be explored further.

Compared to the other studies which assessed disease progression after prior endocrine therapy, including CONFIRM study (CBR: 45.6 %; PFS: 6.5 months)^15^, ^36^, FINDER2 study (47.8%; 6.0 months)^12^, and China COMFIRM study (48%; 8.0 months)^37^, the median PFS of Fulvestrant second-line users (69.9%; 11.2 months) in our study showed equivalent or even better outcomes and discrepancy observed could be due to the difference in sensitivity to endocrine therapy. And also, nearly half or more of the enrolled patients in CONFIRM and FINDER2 study showed relapsed during adjuvant endocrine therapy, whereas in our study, 69 (22.8%) patients were primary resistant to endocrine therapy. However, this inference was consistent with clinical observation, but need further research and verification. In addition, results show second-line patients treated with Fulvestrant with chemotherapy or endocrine therapy in the first-line did not significantly affect the efficacy of Fulvestrant. This further suggests that, second-line use of Fulvestrant was also an effective regimen after chemotherapy.

Furthermore, we analyzed the specific factors affecting the efficacy of the patients with visceral metastases. Generally, breast cancer patients with visceral crisis or symptomatic visceral metastases requires prioritization of chemotherapy for rapid disease relief. However, in presence chemotherapy intolerance or asymptomatic visceral metastases, endocrine therapy was still an optional treatment. Previous studies^17^, ^21^ have demonstrated no significant advantage over anastrozole in Fulvestrant users with visceral metastases. Similarly, in our study the PFS in patients with visceral metastases was significantly shorter despite receiving Fulvestrant as first-line endocrine therapy (*p*=0.021). For patients with visceral metastases, the multivariate COX analyses revealed that liver metastases (*p*=0.001) was a significant risk factor for Fulvestrant treatment, while presence of lung metastases made no significant difference (*p*=0.266). This result suggested that the visceral metastases, especially liver metastases, was a poor prognostic factor for Fulvestrant users. Similar findings were observed in another study with liver metastases had significantly poorer response in Fulvestrant treatment than patients with visceral non-liver metastases^38^. Our study findings also revealed that, long disease-free survival and less metastatic sites were positive predictors for PFS of Fulvestrant users with visceral metastases.

Current clinical data for premenopausal patients was limited and guidelines for treatment decisions are often made in reference to the evidence of postmenopausal patients. The median PFS reported in phase 3 randomized controlled trials which contains Fulvestrant plus GnRHa (gonadotropin‐releasing hormone agonist) arm were 4.6 months (PALOMA-3) and 9.3 months (MONARCH‐2) 39, 40. In our study, 48 patients had premenopausal status and reached postmenopausal status through drug-induced ovarian function suppression. However, no significant difference in the efficacy of Fulvestrant treatment between these drug-induced menopausal and natural menopausal patients was observed (6.3 vs 8.9 months, *p*=0.739). This illustrates that Fulvestrant treatment can be an effective choice for premenopausal patients with drug-induced menopause.

Given the definition of subgroups from 15^th^ St. Gallen International Breast Cancer conference 2017, there was no absolute boundary between the luminal A-like and the luminal B-like^41^. Current trends consider hormone receptor, proliferation index, grade as factors for judgement. In our study, we found that the level of hormone receptor expression and proliferation index were not significant related to the efficacy of Fulvestrant. This suggested that similar efficacy can be obtained with Fulvestrant in patients with luminal A-like or B-like.

With the recent emergence of cyclin-dependent kinase 4 and 6 inhibitors, the encouraging outcome of PFS even OS has made the combination therapy a considerable option for primary treatment of metastatic breast cancer. However, it accompanies with high financial burden and increased toxicity accordingly. Hence, Fulvestrant as primary endocrine therapy can be a preferred choice for selected patients with long disease-free interval, light tumor burden and less palliative chemotherapy before Fulvestrant.

This study has a relatively large sample size and features consistent with the Chinese breast cancer population^25^, ^26^, the results can better reflect the actual situation of Chinese patients using Fulvestrant than the existing real-world studies in this direction. Our study has few limitations. Firstly, this was a single-center retrospective study. Secondly, there was no comparative arm to ensure Fulvestrant performance with other agents. And lastly, drug-resistance problem like other endocrine therapy was not assessed in our study. However, the main strength of our retrospective real-world study was the implication of this study results for both clinical practice and research.

## Conclusion

This study demonstrated that HR+/HER2˗ real-world advanced breast cancer patients receiving Fulvestrant as first-line treatment respond significantly better than higher-line treatment with significantly longer PFS for the patients with light tumor burden, less palliative chemotherapy before Fulvestrant and long disease-free interval. For patients receiving Fulvestrant after palliative chemotherapy, the median PFS was numerically greater in maintenance treatment group than those who progressed after chemotherapy. The premenopausal patients receiving Fulvestrant combined with ovarian function suppression can obtain similar efficacy as postmenopausal patients. Patients of different luminal subtypes can also benefit from Fulvestrant. For patients with visceral metastases, presence of liver metastases was a significant risk factor on effect of Fulvestrant treatment, while presence of lung metastases made no significant difference.

## Figures and Tables

**Table 1:** Demographic and disease characteristics

Patients Characteristics	n	%
Age (years)		
Median (range)	51 (21-82)	
Menopause status at Fulvestrant usage		
Premenopausal^#^	48	15.8
Postmenopausal	255	84.2
Histologic type		
IDC	235	77.6
Others	68	22.4
HR status		
ER and/or PgR positive		
1-10%	22	7.3
11-25%	34	11.2
26-50%	20	6.6
51-100%	213	70.3
Unknown	14	4.6
Ki67 index		
0	1	0.3
1-20%	106	35.0
>20%	120	39.6
Unknown	76	25.1
Stage of disease		
Primary stage IV	39	12.9
Recurrence	264	87.1
Adjuvant chemotherapy*		
Anthracycline and taxanes	77	29.2
Anthracycline only	49	18.6
Taxanes only	38	14.4
Other regimens	43	16.3
No adjuvant chemotherapy	37	14.0
Unknown	20	7.6
Adjuvant endocrine therapy*		
Yes		
SERMs (tamoxifen and/or toremifene)		
< 5 years	59	22.3
≥ 5 years	43	17.8
SERMs followed by aromatase inhibitors		
< 5 years	11	4.2
≥ 5 years	12	4.5
AIs		
<5 years	50	18.9
≥ 5 years	37	14.0
No	43	16.3
Unknown	9	3.4
Adjuvant radiotherapy*		
Yes	109	41.3
No	129	48.9
Unknown	26	9.8
DFS*		
< 1 year	11	4.2
1-2 years	17	6.4
2-3 years	34	12.9
3-5 years	56	21.2
5-10 years	96	36.4
> 10 years	50	18.9

IDC: invasive ductal carcinoma; HR: hormone receptor; ER: estrogen receptor; PgR: progesterone receptor; SERMs: selective estrogen receptor modulator; AIs: aromatase inhibitors; DFS: disease-free survival. ^#^Premenopausal patients received ovarian function suppression in duration of Fulvestrant treatment. *Patients with primary stage IV disease were excluded (n=39).

**Table 2:** The status of metastases and treatment

Variables	n	%
Visceral disease at Fulvestrant		
Yes	207	68.3
No	96	31.7
Metastatic site		
Bone	219	72.3
Lymph node/soft tissue	216	71.3
Liver	82	27.1
Lung	137	45.2
Brain	13	4.3
Bone only	37	12.2
Number of metastatic sites		
1-2	148	48.8
3-4	119	39.3
≥ 5	36	11.9
Previous chemotherapy for metastatic breast cancer		
None	106	35.0
1 or 2 lines	158	52.1
≥ 3 lines	39	12.9
Previous endocrine therapy for metastatic breast cancer		
SERMs	7	2.3
AIs	200	66.0
none	96	31.7
SERMs before Fulvestrant		
For adjuvant ET only	104	34.3
For MBC	61	20.1
No	123	40.6
Unknown	15	5.0
AIs before Fulvestrant		
For adjuvant ET only	66	21.8
For MBC	199	65.7
No	32	10.6
Unknown	6	2.0
Fulvestrant at treatment line		
First-line	53	17.5
Second-line	73	24.1
≥ third-line	177	58.4
Fulvestrant at ET line		
First-line ET	96	31.7
Second-line ET	123	40.6
≥ third-line ET	84	27.7
Fulvestrant at first-line ET*		
Without chemotherapy	53	55.2
Progression after chemotherapy	29	30.2
Maintenance treatment after chemotherapy	14	14.6
Endocrine therapy sensitivity		
Sensitive	69	22.8
Primary resistance	82	27.1
Secondary resistance	152	50.2

SERMs: selective estrogen receptor modulator; AIs: aromatase inhibitors; ET: endocrine therapy; MBC: metastatic breast cancer. *Numbers of patients received Fulvestrant as first endocrine therapy was 96 in total.

**Table 3:** Univariate and multivariate analysis of factors for progression-free survival

Variables		Univariate analysis		Multivariate analysis	
	n	Median (95% CI)	*p-*Value	Exp (B) (95% CI)	*p-*Value
Age at Fulvestrant					
< 50	68	5.4 (3.7-7.1)	**0.004**		0.622
≥ 50	235	9.4 (7.8-11.0)			
Menopause status at Fulvestrant					
Premenopausal^#^	48	6.3 (3.4-9.2)	0.140		0.739
Postmenopausal	255	8.9 (7.4-10.5)			
Status of disease					
Stage IV	39	8.9 (4.4-13.4)	0.299		
Recurrence	264	8.4 (6.9-10.0)			
Histologic type					
IDC	235	8.5 (7.0-10.0)	0.728		
None IDC	55	8.1 (2.7-13.4)			
HR status					
≤ 50%	76	7.4 (5.5-9.4)	0.550		
> 50%	213	9.1 (7.2-11.0)			
Ki67 index					
1-20%	106	8.1 (6.3-9.9)	0.809		
> 20%	120	8.3 (4.5-12.2)			
DFS					
< 2 years	28	5.8 (3.9-7.7)	0.106		**0.027**
2-5 years	34	5.7 (1.1-10.2)		1.48 (0.87-2.51)	0.144
> 5 years	202	9.4 (7.2-11.6)		1.90 (1.18-3.06)	0.009
Visceral metastases at Fulvestrant					
Yes	207	7.2 (5.6-8.9)	**<0.001**		0.064
No	96	13.4 (10.0-16.9)			
Number of metastatic sites					
1-2	148	12.6 (8.2-17.1)	**<0.001**		**<0.001**
3-4	119	7.7 (5.8-9.6)		0.27 (0.16-0.44)	<0.001
≥ 5	36	4.0 (2.9-5.1)		0.41 (0.25-0.66)	<0.001
Previous chemotherapy for metastatic breast cancer					
None	106	14.1 (7.1-21.0)	**<0.001**		**0.003**
1 or 2 lines	158	8.0 (5.1-10.8)		0.39 (0.23-0.66)	<0.001
≥ 3 lines	39	3.5 (2.8-4.1)		0.56 (0.35-0.89)	0.013
Liver metastases					
Yes	82	4.6 (3.1-6.1)	**<0.001**		
No	221	12.5 (9.6-15.4)			
Lung metastases					
Yes	137	8.9 (7.7-10.1)	0.734		
No	166	8.4 (5.5-11.4)			
Brain metastases					
Yes	13	5.1 (2.8-7.3)	0.051		
No	290	8.9 (7.5-10.4)			
Previous ET for metastatic breast cancer					
SERMs	7	6.5 (1.2-11.8)	0.298		
AIs	200	7.8 (6.1-9.5)			
None	96	10.5 (6.5-14.6)			
Fulvestrant at treatment line					
First-line	53	14.1 (10.1-18.0)	**0.002**		0.148
Second-line	73	11.2 (2.2-20.3)			
≥ third-line	177	6.7 (4.8-8.5)			
Fulvestrant at ET line					
First-line ET	96	10.5 (6.5-14.6)	0.207		
Second-line ET	123	7.7 (5.7-9.7)			
≥ third-line ET	84	8.1 (5.0-11.1)			

CI: confidence interval; IDC: invasive ductal carcinoma; HR: hormone receptor; DFS, disease-free survival; ET: endocrine therapy; SERMs: selective estrogen receptor modulator; AIs: aromatase inhibitors. ^#^Premenopausal patients received ovarian function suppression in duration of Fulvestrant treatment.

**Table 4a:** Univariate and multivariate analysis for progression-free survival of Fulvestrant used in primary endocrine treatment line

Variables		Univariate analysis			Multivariate analysis	
	n	Median (95% CI)	*p-*Value		Exp (B) (95% CI)	*p-*Value
Age at Fulvestrant						
< 50	21	7.5 (2.5-12.3)	0.096			0.124
≥ 50	75	12.5 (7.4-17.5)				
Visceral metastases at Fulvestrant						
Yes	59	8.3 (5.8-10.8)	**0.005**		0.45 (0.23-0.89)	**0.021**
No	37	NR				
Number of metastatic sites						
1-2	61	15.9 (8.0-23.8)	0.088			0.915
3-4	26	9.2 (3.8-14.6)				0.747
≥ 5	9	4.0 (0.7-5.3)				0.675
Liver metastases						0.831
Yes	21	7.0 (1.0-13.0)	**0.022**			
No	75	13.4 (6.5-20.4)				
Previous chemotherapy for metastatic breast cancer						
None	53	14.1 (10.1-18.0)	**0.004**			**0.008**
1-2	40	5.8 (2.1-9.4)			0.06 (0.01-0.28)	<0.001
≥ 3 lines	3	2.4 (0.1-4.7)			0.08 (0.02-0.39)	0.002
Endocrine therapy sensitivity						
Sensitive	9	30.8 (1.0-60.6)	**<0.001**			**<0.001**
Primary resistance	29	3.9 (3.5-4.3)			0.17 (0.04-0.76)	0.02
Secondary resistance	58	14.1 (9.2-19.0)			5.18 (2.39-11.19)	<0.001
Fulvestrant after CT*						
Progression after CT	29	4.9 (2.5-7.2)	0.255			
Maintenance treatment after CT	14	10.6 (0.0-22.4)				

CI: confidence interval; CT: chemotherapy. *Number of patients using Fulvestrant in first-line endocrine therapy after chemotherapy was 43.

**Table 4b:** Univariate and multivariate analysis for progression-free survival of visceral metastasis

Variables		Univariate analysis			Multivariate analysis	
	n	Median (95% CI)	*p-*Value		Exp (B) (95% CI)	*p-*Value
Age at Fulvestrant						
< 50	48	4.9 (2.1-7.7)	**0.026**			0.612
≥ 50	159	7.8 (6.0-9.6)				
Menopause status at Fulvestrant						
Premenopausal^#^	34	5.1 (2.2-7.9)	0.18			0.763
Postmenopausal	173	7.7 (6.1-9.3)				
DFS						
< 2 years	16	5.8 (3.6-8.1)	**0.007**			**0.005**
2-5 years	26	3.5 (1.1-6.0)			1.31 (0.67-2.54)	0.427
> 5 years	139	8.1 (6.3-9.9)			2.52 (1.51-4.22)	<0.001
Number of metastatic sites						
1-2	65	8.9 (3.9-13.9)	**0.005**			**0.004**
3-4	106	7.7 (5.6-9.9)			0.38 (0.22-0.66)	0.001
≥ 5	36	4.0 (2.9-5.1)			0.53 (0.32-0.88)	0.014
Previous chemotherapy for metastatic breast cancer						
None	64	9.4 (3.9-14.9)	**0.001**			0.064
1 or 2 lines	112	5.7 (3.9-7.4)				
≥ 3 lines	31	3.5 (1.9-5.1)				
Liver metastases						
Yes	82	4.6 (3.1-6.1)	**<0.001**		0.52 (0.35-0.78)	**0.001**
No	125	9.3 (5.4-13.2)				
Lung metastases						
Yes	137	8.9 (7.7-10.1)	**0.008**			0.266
No	70	4.7 (3.6-5.9)				
Brain metastases						
Yes	13	5.1 (2.8-7.3)	0.175			0.664
No	194	7.7 (6.2-9.3)				
Bone metastases						
Yes	149	6.2 (4.6-7.9)	**0.031**			0.423
No	58	10.6 (5.2-15.9)				

CI: confidence interval; DFS: disease-free survival. ^#^Premenopausal patients received ovarian function suppression in duration of Fulvestrant treatment.

**Table 5:** Summary of adverse events

Adverse events	Grade 1	Grade 2	Grade 3	Grade 4
Fatigue	1	1		
Oral ulcer	1			
Leukocyte decrease	1			
Blood bilirubin increase		1		
Thromboembolic events			1	
Alanine aminotransferase increased	1	1		
Headache	2			
Palpitation	2			
Hyperglycemia	1			
Anorexia	1			
Backache	1			
